# Side effect management

**DOI:** 10.1186/1471-227X-15-S1-A6

**Published:** 2015-06-24

**Authors:** Tommaso Pellis

**Affiliations:** 1 Department of Anesthesia and Intensive Care, Santa Maria degli Angeli Hospital, Pordenone, Italy

## 

Therapeutic hypothermia (TH) induces a paraphysiological state that can determine clinical modifications which do not constitute necessarily adverse effects, thus warranting monitoring rather than over corrective action in most circumstances. Others are known side effects related to hypothermia and can be anticipated.

Rapid induction of hypothermia by infusion of cold crystalloids with a pressure bag (2 l at 300 mmHg) is associated with an increase rate of pulmonary edema and rearrests [1].

The Hypothermia Network Registry reports adverse events of 986 patients treated with TH [2]. Bradycardia was the most common arrhythmia (13%), but pacing was very rarely necessary, suggesting that bradycardia should be tolerated rather than treated if hemodynamic stability is preserved. Pneumonia was the most frequent infection (41%).

Bleeding requiring transfusion occurred in 4%; the risk was significantly higher if coronary angiography was performed (2.8% vs. 6.2%, *P* = 0.02). Sustained hyperglycemia (8 mmol >4 hours) was observed in 37% of patients. Electrolyte disorders were also quite common (18%): specifically hypokalemia, hypomagnesemia, and hypophosphatemia. These derangements should be anticipated, tightly monitored and promptly corrected. The incidence of sepsis was only 4% but was higher in patients with: intravascular devices for temperature management (OR 2.6), intraortic balloon pump (OR 3.2) or undergoing coronary angiography (OR 4.4). Yet infection, bleeding, arrhythmia and electrolyte disorders were not associated with increased mortality.

The Target Temperature Management Trial – the largest trial on temperature management – reported the rate of adverse events as one of the secondary outcomes [3]. There was no difference in adverse events between the 33°C and 36°C arms (respectively 93% vs. 90%, *P*= 0.086). Among the over 30 subtypes, bleeding, particularly in critical sites, was extremely rare. Bleeding was most common from the insertion sites (33°C, 9.2% vs. 36°C, 6.1%, *P*= 0.076). Again pneumonia was the most common infection regardless the level of hypothermia (52% vs. 46%, *P* = 0.089). The incidence of bradycardia requiring pacing was low and equal in the two groups, suggesting that hypothermia is not responsible for hemodynamically compromising bradycardia (5.2% vs. 6.4%). The only subgroup with a significantly different rate was hypokalemia (19% vs. 13%, *P* = 0.018). Hypomagnesemia and hypophosphatemia did not differ but were frequent (respectively 20% and 41%), highlighting the need to anticipate electrolyte imbalances.

In conclusion, adverse events can be anticipated and managed. The incidence is not related to the level of temperature management and is not associated with worse survival or neurological recovery.

## Financial disclosure

TP has received speaker’s reimbursement from C. R. BARD.

## Declaration

This abstract and supplement was proposed, developed and commissioned by BMC Emergency Medicine and was funded by an educational grant from C. R. BARD, NJ, USA. The published abstract was independently prepared by the author. C. R. BARD had no input into the content.
